# Diagnostic variability in sarcopenia and sarcopenic obesity among older adults: a clinical and public health challenge

**DOI:** 10.3389/fnut.2026.1895826

**Published:** 2026-08-27

**Authors:** Táscya Morganna de Morais Santos, Gabriel Soares Bádue, Nykholle Bezerra Almeida, Celina de Azevedo Dias, Jessiane Rejane Lima Santos, Piettra Moura Galvão Pereira, Enaiane Cristina Menezes, Fabiana Andréa Moura, João Araújo Barros-Neto

**Affiliations:** 1 Faculty of Nutrition, Federal University of Alagoas, Maceió, Brazil; 2 Institute of Biological and Health Sciences, Federal University of Alagoas, Maceió, Brazil; 3 Institute of Physical Education and Sports, Federal University of Alagoas, Maceió, Brazil

**Keywords:** concordance, older adults, prevalence, sarcopenic, sarcopenic obesity

## Abstract

**Introduction:**

Sarcopenia and sarcopenic obesity (SO) are highly prevalent among older adults; however, the lack of standardized diagnostic criteria compromises epidemiological comparability and clinical practice. Therefore, this study aimed to evaluate differences in the prevalence and diagnostic agreement of sarcopenia and SO according to different indicators of muscle strength and adiposity.

**Methods:**

A cross-sectional study was conducted among 870 community-dwelling older adults. The prevalence and diagnostic agreement of sarcopenia and sarcopenic obesity (SO) were compared using different combinations of indicators of muscle strength (handgrip strength and the sit-to-stand test), muscle mass (fat-free mass index), and adiposity (BMI, waist circumference, and body fat percentage).

**Results:**

The prevalence of sarcopenia ranged from 9.2% (95% CI = [7.40–11.36]) to 18.8% (95% CI = [16.30–21.65]) and was significantly higher when the sit-to-stand test was used as the indicator of muscle strength (*p* < 0.001). The prevalence of SO ranged from 0 to 5.17% (95% CI = [3.84–6.92]; *p* < 0.001), with lower estimates when BMI was used as the obesity criterion (0.23%; 95% CI = [0.04–0.92]) and 0.46% (95% CI = [0.15–1.26]), and higher prevalence when body fat percentage was used (3.22%; 95% CI = [2.19–4.68]) and 5.17% (95% CI = [3.84–6.92]). Diagnostic agreement ranged from “no agreement” to “substantial agreement,” with the highest agreement observed between criteria using body fat percentage (*κ* = 0.62; 95% CI = [0.47–0.74]).

**Conclusion:**

The findings demonstrate substantial variability in the prevalence of sarcopenia and SO according to the diagnostic criteria adopted, reinforcing the need to standardize more sensitive and consistent methods for population screening and clinical decision-making among older adults.

## 1 Introduction

Sarcopenia and sarcopenic obesity (SO), syndromes characterized by the progressive loss of muscle strength, mass, and function, with high prevalence among older adults, are associated with adverse clinical outcomes such as increased risk of falls, functional decline, frailty, and reduced life expectancy (1). Defined by the coexistence of excess adiposity and sarcopenia, SO further increases the risk of cardiovascular disease and other chronic noncommunicable diseases to these clinical complications, thereby negatively affecting health-related quality of life (2), and establishing SO as one of the poorest prognostic scenarios in geriatric care. Consequently, this condition extends beyond the challenge of individual clinical management, becoming a major public health concern with direct implications for the sustainability and costs of public healthcare systems, particularly in light of the rapid pace of population aging worldwide.

The global prevalence of sarcopenia among community-dwelling older adults, that is, individuals who are neither hospitalized nor institutionalized in long-term care facilities, is estimated to range from 9.9 to 40.4% (3–5), whereas the prevalence of SO in this same age group ranges from 2 to 25% (6–9). This substantial epidemiological variability reflects not only the demographic and ethnic heterogeneity of these populations, but, more importantly, differences in the diagnostic criteria and cutoff points adopted for diagnosis (10, 11). The lack of universal methodological standardization creates a scenario of uncertainty that compromises data comparability and scientific reproducibility (3, 12–14). In clinical practice, this inconsistency hinders the development of precise therapeutic guidelines and the establishment of accurate reference values, limiting the ability of healthcare professionals to intervene early in promoting active and healthy aging.

Although different international consensuses and consortia generally share muscle strength, muscle mass, and physical function as fundamental domains for the diagnosis of these conditions, these documents often diverge regarding the criteria, approaches, and cutoff points adopted (1, 2, 15–17). Although these initiatives have been fundamental to the early advances in the diagnosis of these conditions, the persistence of methodological diversity limits their uniform applicability in population-based studies, as even the adoption of a single consensus may generate substantial variation in prevalence estimates within the same community.

In this context, refining the parameters used to assess muscle function, particularly muscle strength and muscle mass, as well as establishing more standardized criteria for classifying adiposity, constitute a fundamental step toward improving the accuracy of sarcopenia and SO diagnosis. Such improvements may contribute to a more accurate epidemiological understanding of conditions associated with alterations in body composition while also supporting clinical and nutritional care strategies at both the individual and population levels. Thus, defining a diagnostic standard that is simultaneously sensitive, specific, and accessible to healthcare services remains one of the greatest challenges for the comprehensive care of older adults.

In light of the foregoing, this study aimed to examine differences in the estimated prevalence of sarcopenia and SO based on combinations of different indicators of muscle strength and adiposity recommended for the diagnosis of these clinical conditions, as well as the level of diagnostic agreement among these measures in a representative sample of community-dwelling older adults, thereby reinforcing the urgent need to establish more precise diagnostic criteria.

## 2 Methods

### 2.1 Study design

A cross-sectional study was conducted using secondary data from the “First Alagoas Diagnosis of Health, Nutrition, and Quality of Life of Older Adults,” carried out between April 2022 and January 2024 and reported in accordance with using the Strengthening the Reporting of Observational Studies in Epidemiology (STROBE) statement.

### 2.2 Ethical aspects

The study was conducted in accordance with the principles established in Resolutions No. 466/2012 and No. 510/2016 of the Brazilian National Health Council. The study was approved by the Research Ethics Committee of the Federal University of Alagoas, Brazil, under CAAE No. 39960320.2.0000.5013 and approval opinion No. 4.665.172/2021. All participants provided written informed consent form.

### 2.3 Sample, sampling and eligibility criteria

A representative sample of community-dwelling older adults living in the state of Alagoas was calculated based on the estimated population aged 60 years and older. To accomplish this, the sampling strategy adopted the protocol established for the parent study and was implemented using a three-stage design. First, cluster sampling was performed by randomly selecting five municipalities from each of the two Health Macroregions of Alagoas, together with the state capital, Maceió, resulting in a total of 11 municipalities (Maceió, Arapiraca, Campo Alegre, Flexeiras, São Miguel dos Campos, Santana do Ipanema, Pariconha, Pão de Açúcar, Pilar, São Sebastião, and União dos Palmares). Next, two census tracts were randomly selected within each municipality. In the final stage, a random starting point was established in each selected census tract, from which households were systematically sampled. The number of households included in each municipality was proportional to the size of its older adult population.

The final sample size was determined based on the number of older adults residing in the state of Alagoas, considering the known finite population of the state. A confidence level of 99% and a maximum margin of error of 2% were adopted for the sample size calculation. The minimum sample required for the parent study was estimated at 955 participants. To account for potential refusals and participant losses, an additional 20% was incorporated into the calculation, resulting in an expected minimum sample of 1,147 community-dwelling older adults. The parent study ultimately enrolled 1,153 participants, exceeding the minimum required sample size. For the present study, individuals aged ≥60 years, of both sexes, who were physically able to attempt bioelectrical impedance analysis, waist circumference measurement, handgrip strength assessment, and the five-times sit-to-stand test were included. Individuals unable to safely perform any of these procedures (e.g., due to severe mobility impairment preventing test execution) were excluded. It is important to note that the ability to attempt the five-times sit-to-stand test was the eligibility requirement, not the time taken to complete it. Test performance was subsequently classified according to the standard cutoff, with a completion time >15 s indicating poor muscle function, consistent with sarcopenia/dynapenia diagnostic criteria. Accordingly, the final sample consisted of 870 individuals.

### 2.4 Data collections and variables

Data collection was carried out by researchers (undergraduate and graduate students in the Nursing, Physical Education, Pharmacy, Medicine, Nutrition, and Psychology programs), who were properly trained and qualified for the study under the supervision of faculty researchers from the Federal University of Alagoas. Data were collected through household visits using a previously established and validated structured protocol containing fields for sociodemographic, economic, past and current health, and lifestyle variables. Subsequently, body composition assessments and functional performance tests were conducted. The variables included in the data collection protocol are described below.

#### 2.4.1 Variables related to sociodemographic, economic, health, lifestyle, and functional capacity conditions

Identification data were collected, including name, sex, age, and race/skin color. Age was categorized as <80 years (non-long-lived older adults) and ≥80 years (long-lived older adults). Race/skin color [other races (White, Asian, Indigenous) and black individuals (black, mixed race)]. Participants who self-identified as White, Asian, or Indigenous were grouped into the “Other races” category to avoid categories with low frequencies, particularly among Asian and Indigenous participants, and to ensure greater stability of the statistical analyses. Marital status was categorized as without a partner (single, widowed, or divorced) and with a partner (married or in a stable union). Educational level was categorized as <4 years and ≥4 years of schooling, and area of residence was classified as “urban” or “rural.” Monthly household income was categorized as ≤1 minimum wage and >1 minimum wage.

The occurrence of previously diagnosed noncommunicable diseases (NCDs) selected for this study was determined through self-report and subsequently confirmed by reviewing participants’ medical records available in the electronic health record system of Brazil’s Unified Health System (SUS), as well as by the presentation of a valid medical prescription for the continuous use of medications indicated for the treatment of these conditions. Only NCDs listed in the Strategic Action Plan to Tackle Chronic Diseases and Non-Communicable Diseases in Brazil, 2021–2030, were considered. The conditions included arterial hypertension, diabetes mellitus, cardiovascular diseases (CVD), rheumatism, and osteoporosis (18). Alcohol consumption and smoking status were assessed based on affirmative or negative responses regarding the consumption of any amount of alcoholic beverages and the use of cigarettes, tobacco products, related products, and similar substances at least once within the previous 3 months. Physical activity level was assessed using the International Physical Activity Questionnaire (IPAQ) and classified according to its criteria as active or insufficiently active (19). Regarding functional capacity, older adults were classified as “dependent” or “independent” according to their ability to perform Basic Activities of Daily Living (BADL), assessed using the Katz Index, which measures functional independence in personal care, mobility, locomotion, and physiological eliminations (urination and defecation), with individuals classified as dependent if they presented limitations in at least one BADL (20). The Timed Up and Go (TUG) test and Gait Speed (GST) Test were also performed. For the TUG test, older adults were instructed to stand up from a chair, walk three meters, return, and sit down again in the same chair. Performance time of ≥20 s was considered impaired (1). For the GST, the time required for older adults to walk 4.6 meters at their usual walking pace was recorded. Three trials were performed, and the final value corresponded to the mean of these measurements. A gait speed ≥ 0.8 m/s was considered low. Data regarding medication use were also collected, and polypharmacy was defined as the concurrent use of five or more medications, with or without a prescription (21). Information regarding the occurrence of falls within the previous year was obtained by self-reported.

#### 2.4.2 Assessment of low muscle strength

##### 2.4.2.1 Handgrip strength (upper limbs)

As the first criterion, low muscle strength was assessed using the handgrip strength (HGS) test with a hydraulic hand dynamometer. Participants were instructed to squeeze the dynamometer with their dominant hand using maximal force. Grip force was maintained for 5 s during each of the three trials, and the highest value obtained was used for analysis. Cutoff points of <27 kg for men and <16 kg for women were adopted to classify low muscle strength (1). The inability to perform the handgrip strength test was associated with joint conditions of the hands or wrists.

##### 2.4.2.2 Five-times sit-to-stand test

The sit-to-stand (STS) test was also performed using an armless, wheelless chair with a straight backrest positioned against a wall. Participants were instructed to cross their arms over their chest and to stand up and sit down five times as quickly and safely as possible, without using their hands to assist the movement. The shortest time recorded was used for analysis. A time of <15 s was classified as ‘sufficient’ strength. Older adults who completed the test in more than 15 s were classified as having ‘insufficient’ strength (1). The inability to perform the sit-to-stand test was primarily attributed to knee or hip joint conditions (osteoarthritis, deformities, or recent surgery).

#### 2.4.3 Assessment of low muscle mass

Low muscle mass was identified through indirect assessment using the Fat-Free Mass Index (FFMI) (22). Fat-free mass was assessed using a tetrapolar bioelectrical impedance analysis (BIA) device (Tanita Corporation, model BC601GTM, Tokyo, Japan). The FFMI was calculated by dividing whole-body fat-free mass by height squared (FFM/height^2^). Low muscle mass was defined using FFMI cutoff points of <18 kg/m^2^ for men and <15 kg/m^2^ for women (22).

#### 2.4.4 Anthropometric measures and indices

Body weight was measured during the BIA assessment, with participants barefoot, wearing as little clothing as possible, and standing upright. With the head positioned in the Frankfurt plane, shoulders relaxed, and arms extended alongside the body (23). Height was measured with participants barefoot, standing upright with their feet together and their heels, buttocks, and head in contact with a portable stadiometer (Seca®), graduated in tenths of a centimeter and positioned on a flat surface (24). Body Mass Index (BMI) was calculated as the ratio between body weight (kg) and height squared (m^2^). Classification followed the criteria established by the Pan American Health Organization, which defines obesity as a BMI ≥ 30 kg/m^2^ (25).

Waist circumference was measured using a flexible, nonelastic measuring tape positioned around the abdomen without compression. The measurement was performed at the midpoint between the inferior margin of the last palpable rib and the top of the iliac crest. Participants remained standing upright, with their arms relaxed at their sides and body weight evenly distributed between both feet. The assessment was recorded at the end of a normal expiration. Two waist circumference measurements were obtained, and the mean value was calculated. If the difference between the two measurements exceeded 1 cm, the measurement was repeated and the previous measurements were discarded. The cutoff points for excess adiposity were >102 cm for men and >88 cm for women (26).

#### 2.4.5 Body composition

Participants were instructed to step onto the BIA platform barefoot, remain still in an upright position, place their heels on the heel electrodes, and raise their arms horizontally with their elbows flexed at a 90° angle while holding the display unit in front of their body. Percentage body fat (%BF) was considered elevated when values exceeded the cutoff points of >27% for men and >38% for women (27).

#### 2.4.6 Diagnosis of obesity

To determine obesity, excess adiposity was assessed using three operational criteria: BMI, waist circumference (WC), and body fat percentage. These indicators were selected because they are recognized as measures of excess adiposity in the European Society for Clinical Nutrition and Metabolism (ESPEN) and the European Association for the Study of Obesity (EASO) consensus framework (2). In the present study, they were applied as alternative operational definitions of obesity in an exploratory approach to examine how the choice of adiposity criterion influences the estimated prevalence of sarcopenic obesity.

#### 2.4.7 Diagnosis of sarcopenia

**Figure 1:**
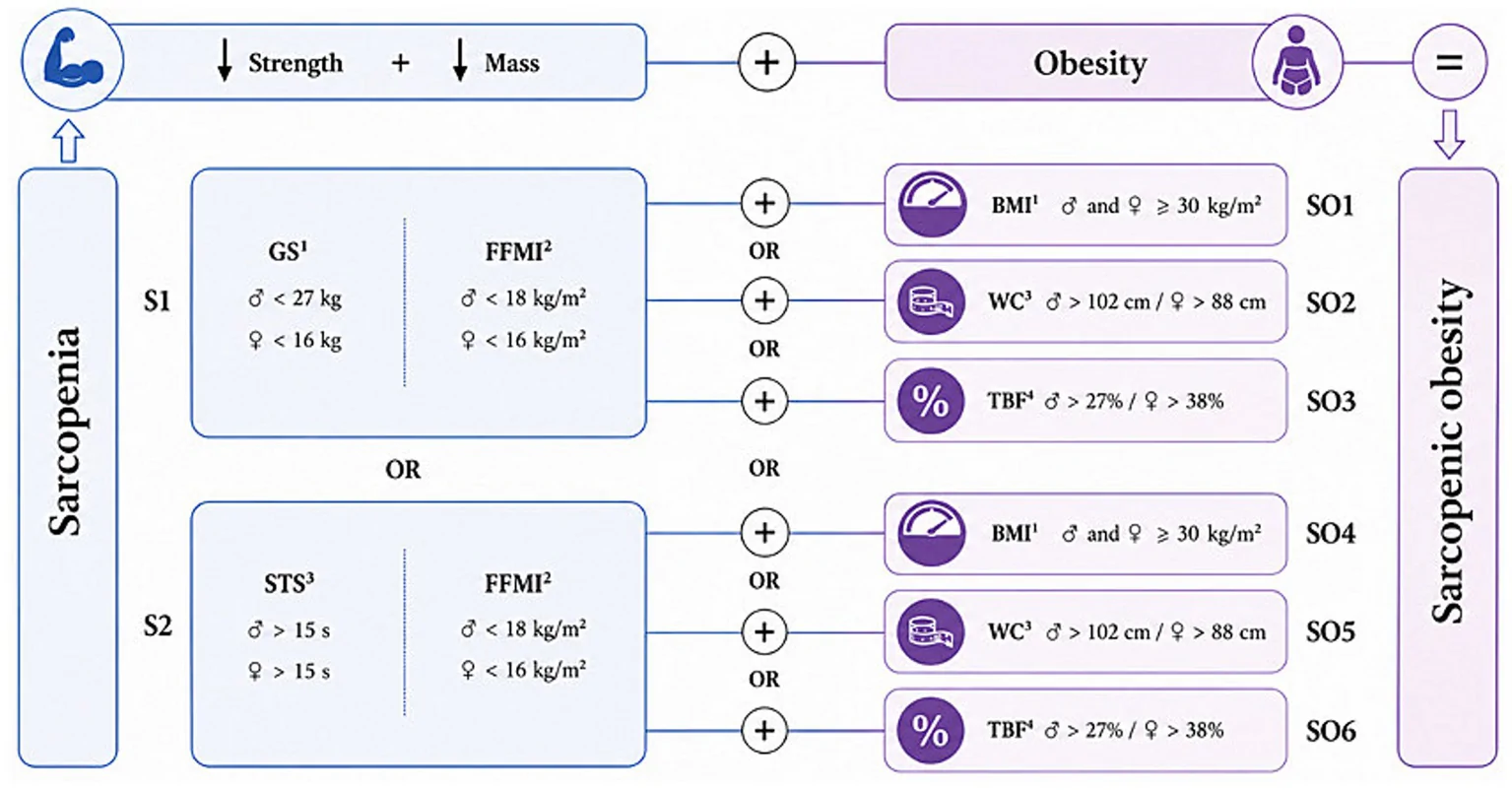
Flowchart of the diagnostic combinations for sarcopenic obesity according to the adiposity and sarcopenia criteria. ^1^Grip strength, ^2^Fat-free mass index, ^3^Sit-to-stand test, ^4^Body mass index, ^5^Waist circumference, ^6^body fat percentage.

Based on the criteria proposed by the European Working Group on Sarcopenia in Older People 2 (EWGSOP2) (1), sarcopenia was diagnosed by the concomitant presence of low muscle strength and low muscle mass. Given the epidemiological nature of this study, which involved a large number of community-dwelling older adults assessed in their homes, the protocol was adapted to ensure its feasibility for field application. Muscle mass was estimated indirectly using the FFMI, as recommended by Kawakami et al. for sarcopenia screening in population-based studies (22), because this was the only method available for all participants. Muscle strength, in turn, was assessed using two different methods: handgrip strength and the five-times sit-to-stand test. Accordingly, two operational definitions of sarcopenia were established: low handgrip strength combined with low muscle mass (S1), and poor performance on the sit-to-stand test combined with low muscle mass (S2), as illustrated in Figure 1.

#### 2.4.8 Diagnosis of sarcopenic obesity (SO)

Sarcopenic obesity was defined by the coexistence of sarcopenia and obesity (2). Rather than applying a single diagnostic algorithm, each adiposity criterion (BMI, WC, and body fat percentage) was independently combined with the sarcopenia criteria to generate alternative operational definitions of sarcopenic obesity. This exploratory strategy was adopted to compare prevalence estimates obtained using different adiposity indicators and to examine the variability introduced by the choice of obesity criterion, rather than to validate or propose new diagnostic definitions.

#### 2.4.9 Combination of different indicators of low muscle strength and obesity for the diagnosis of sarcopenia and sarcopenic obesity

### 2.5 Statistical analyses

After data collection, a database was created using Microsoft Office Excel® version 2019. Subsequently, the R software was used to perform the statistical analyses.

Initially, descriptive analyses were performed using absolute and relative frequencies for categorical variables and means and standard deviations for continuous variables. The prevalence of sarcopenia and sarcopenic obesity was estimated (based on the various combinations of indicators for low muscle strength, low muscle mass, and excess adiposity) as the ratio between the number of individuals classified as having the condition and the total number of participants evaluated. It is worth noting that, since all diagnostic criteria were applied to the same participants and the objective was to compare within-subject diagnostic classifications rather than estimate population differences across municipalities or regions, paired agreement analyses (McNemar’s test, Cochran’s *Q* test, and Cohen’s kappa) were performed using the observed paired classifications. Estimates were expressed as percentages (%) with their respective 95% confidence intervals (95% CI). Subsequently, McNemar’s test was applied to compare the prevalence estimates of sarcopenia. For sarcopenic obesity, Cochran’s *Q* test was used, followed by pairwise analyses using McNemar’s test with Bonferroni adjustment for multiple comparisons. Finally, agreement was assessed using Cohen’s Kappa coefficient, classified as follows: <0.00 poor or no agreement; 0.00–0.20 slight; 0.21–0.40 fair; 0.41–0.60 moderate; 0.61–0.80 substantial; and 0.81–1.00 almost perfect (28). Considering that Cohen’s kappa may be influenced by a marked imbalance in the prevalence of positive and negative classifications, positive percent agreement, and negative percent agreement were also calculated for each pair of diagnostic definitions. Ninety-five percent confidence intervals for Cohen’s kappa, positive percent agreement, and negative percent agreement were estimated using a paired nonparametric bootstrap procedure. When a measure could not be estimated because of empty cells, it was reported as not estimable.

Missing observations resulted from unanswered questions during data collection and were retained as missing values in the database. Analyses were performed using available cases for each variable; consequently, the number of valid observations may differ across variables. A significance level of 5% was adopted for all analyses.

### 2.6 Declaration on the use of artificial intelligence

During manuscript preparation, the authors used the artificial intelligence language tool ChatGPT (OpenAI) to refine the quality of images created by the authors. The artificial intelligence language tool Claude (Anthropic) was used to assist with revisions for style, clarity, objectivity, spelling, and conciseness. It should be emphasized that the entire manuscript was written and critically reviewed by the authors, who remain fully responsible for the factual accuracy, interpretation, and integrity of the final content.

## 3 Results

**Figure 2:**
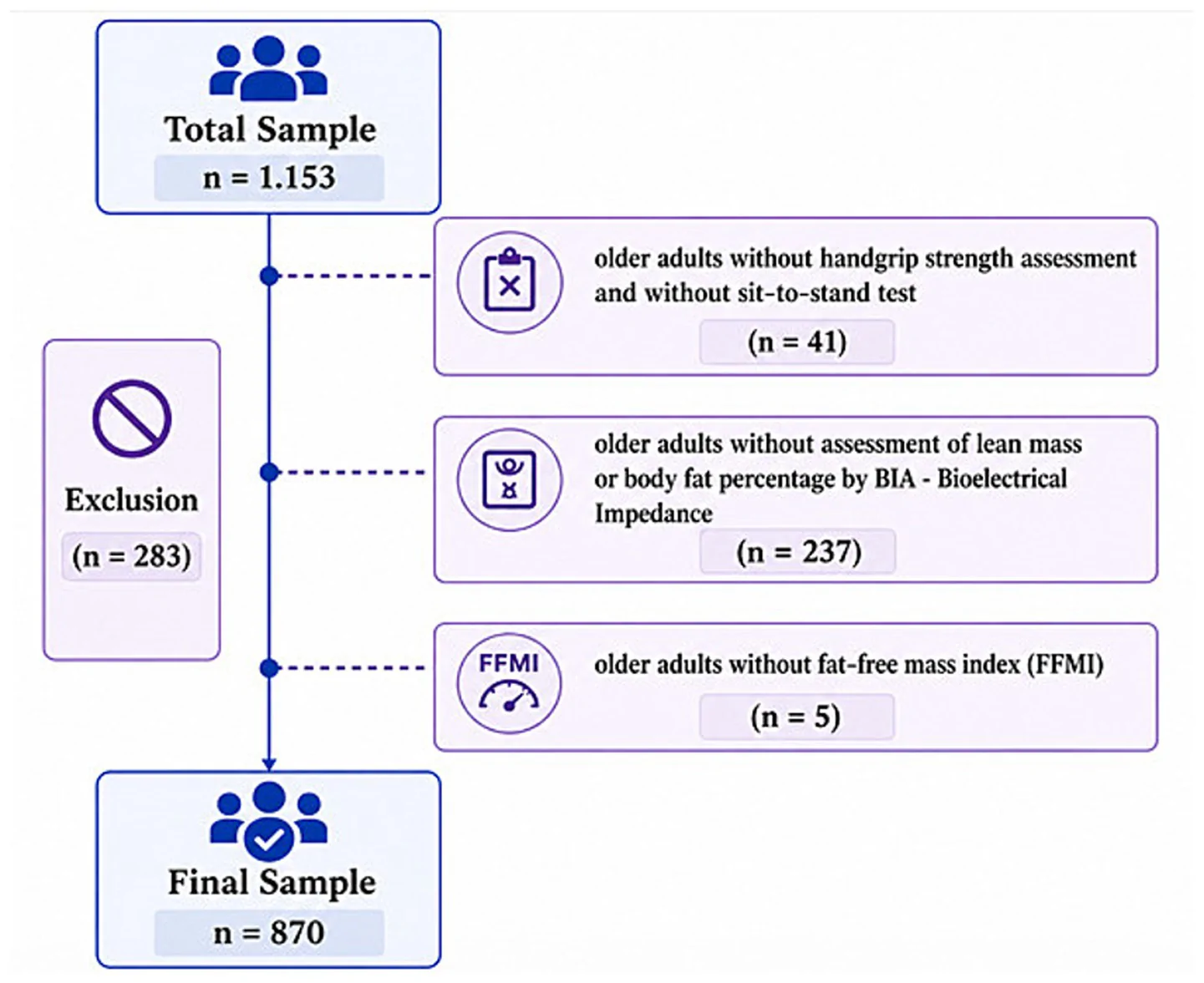
Flowchart of participant recruitment and selection.

Initially, 1,153 individuals were considered eligible for the study; however, 283 were excluded because of physical limitations or missing data, resulting in a final sample of 870 older adults (Figure 2). The mean age of the participants was 70.7 ± 8.13 years (minimum age: 60 years; maximum age: 95 years).

Table 1 presents the characteristics of the sample according to sociodemographic and economic variables, lifestyle, health conditions, and functional capacity. Most participants were female (*n* = 574/870; 66.0%) and non-long-lived older adults (*n* = 728/870; 83.7%). Regarding marital status, nearly 55% were not living with a partner (*n* = 461/844). Regarding race/skin color, more than 73% self-identified as Black or mixed-race (*n* = 639/870), and 73.5% had fewer than 4 years of formal education (*n* = 624/849).

**Table 1:** Sociodemographic, economic, lifestyle, health conditions, and functional characteristics of community-dwelling older adults in the state of Alagoas, 2024.

Variable	Participants	Categories	*n*	%
Sex	870	Male	296	34.0
		Female	574	66.0
Age	870	Non-long-lived older adult	728	83.7
		Long-lived older adult	142	16.3
Marital status	844	With partner	383	45.4
		Without partner	461	54.6
Race/skin color	870	Other race	231	26.6
		Black	639	73.4
Education	849	>4 years	225	26.5
		≤4 years	624	73.5
Area of residence	864	Urban	724	83.8
		Rural	140	16.2
Monthly household income	705	>1 MW	483	68.5
		≤1 MW	222	31.5
Alcohol consumption	866	No	737	85.1
		Yes	129	14.9
Smoking	865	No	726	83.9
		Yes	139	16.1
Physical activity level	834	Physically active	145	17.4
		Insufficiently active	689	82.6
Hypertension	860	No	277	32.2
		Yes	583	67.8
Diabetes	843	No	573	68.0
		Yes	270	32.0
Cardiovascular disease	842	No	730	86.7
		Yes	112	13.3
Rheumatism	829	No	609	73.5
		Yes	220	26.5
Bone diseases	835	No	502	60.1
		Yes	333	39.9
Obesity	866	No	609	70.3
		Yes	257	29.7
Polypharmacy	831	No	711	85.6
		Yes	120	14.4
Falls in the last year	863	None	580	67.2
		1 fall or more	283	32.8
BADL	862	Independent	724	84.0
		Dependent	138	16.0
TUG	650	Normal	343	52.8
		Altered	307	47.2
GST	624	Normal	514	82.7
		Low	110	17.3

MW, Minimum Wage; BADL, Basic Activities of Daily Living; TUG, Timed Up and Go test; GST, Gait Speed Test.

The number of valid observations (*n*) is presented for each variable because missing responses were analyzed as missing values (available-case analysis).

Regarding physical and functional assessment, 16.0% were dependent in basic activities of daily living (BADL) (*n* = 138/862), more than 17% presented low gait speed (*n* = 110/624), and 47.2% showed unsatisfactory performance on the TUG test (*n* = 307/650).

Supplementary Tables S1, S2 present body composition, muscle strength and physical performance variables categorized by sex and age group, respectively.

Heterogeneity was observed in the prevalence estimates of sarcopenia. The prevalence estimated using the S2 combination (18.8%; 95% CI = [16.30, 21.65]) was significantly higher than that observed with the S1 combination (9.2%; 95% CI = [7.40, 11.36]), corresponding to approximately twice as many identified cases (*p* < 0.001) (Table 2). The prevalence of sarcopenic obesity, across the different combinations of criteria employed, also showed substantial variability, ranging from no detected cases in the SO3 combination (0%) to 5.17% (95% CI = [3.84, 6.92]) when the SO6 combination was used as the diagnostic criterion (*p* < 0.001) (Table 2).

**Table 2:** Prevalence of sarcopenia and sarcopenic obesity according to different combinations of criteria in community-dwelling older adults. Alagoas, 2022–2024.

Combinations of diagnostic methods for sarcopenia and sarcopenic obesity (SO)		Prevalence % [95% CI]	*p* ^*^
Sarcopenic	S1	9.20 [7.40, 11.36]	<0.001
	S2	18.8 [16.30, 21.65]	
Sarcopenic obesity	SO1^a^	0.23^d,e,f^ [0.04, 0.92]	<0.001
	SO2^b^	0.46^c,d,e,f^ [0.15, 1.26]	
	SO3^c^	0.00^b,d,e,f^ [0.00, 0.55]	
	SO4^d^	4.71^a,b,c^ [3.44, 6.40]	
	SO5^e^	3.22^a,b,c,f^ [2.19, 4.68]	
	SO6^f^	5.17^a,b,c,e^ [3.84, 6.92]	

a SO1, b SO2, c SO3, d SO4, e SO5, f SO6; superscript letters on each prevalence value indicate a statistically significant difference (McNemar’s test with Bonferroni correction) from the correspondingly lettered combination. BMI, body mass index; WC, waist circumference; %BF (BIA), body fat percentage by bioelectrical impedance analysis; HGS, hand grip strength; STS, sit-to-stand test with time; FFMI, fat-free mass index. Diagnostic combinations: S1 = ↓HGS + ↓FFMI; S2 = ↓STS + ↓FFMI; SO1 = ↑BMI + ↓HGS + ↓FFMI; SO2 = ↑BMI + ↓STS + ↓FFMI; SO3 = ↑WC + ↓HGS + ↓FFMI; SO4 = ↑WC + ↓STS + ↓FFMI; SO5 = ↑BF% + ↓HGS + ↓FFMI; SO6 = ↑BF% + ↓STS + ↓FFMI.

Different letters indicate statistically significant differences among prevalence rates (McNemar test with Bonferroni adjustment for multiple comparisons). ^*^The *p*-value refers to Cochran’s test.

The combinations based on body fat percentage, SO5 (3.22%; 95% CI = [2.19, 4.68]) and SO6 (5.17%; 95% CI = [3.84, 6.92]), showed higher prevalence estimates than those based on BMI, namely SO1 (0.23%; 95% CI = [0.04, 0.92]) and SO2 (0.46%; 95% CI = [0.15, 1.26]) (*p* < 0.001). However, the SO4 combination, which used the STS as the indicator of muscle strength and WC as the adiposity criterion, yielded a prevalence estimates comparable to those obtained with the combinations using body fat percentage as the obesity criterion (4.71%; 95% CI = [3.44, 6.40]), with no statistically significant differences between them.

Across all combinations of criteria evaluated for sarcopenia and sarcopenic obesity, the STS yielded higher prevalence estimates than HGS. In contrast, the lowest prevalence estimates were observed when BMI was used as the adiposity criterion in the diagnostic combinations for sarcopenic obesity (SO1 and SO2), which did not differ significantly from each other and exceeded only the SO3 combination. The SO3 combination, which used elevated waist circumference (WC), low HGS, and reduced FFMI as diagnostic markers, did not identify any cases of sarcopenic obesity in the study population.

Agreement between the different combinations of diagnostic criteria for sarcopenia and sarcopenic obesity is presented in Table 3 and ranged from “no agreement” to “substantial agreement.” Agreement between the diagnostic methods for sarcopenia was moderate (κ = 0.48; 95% CI = [0.39, 0.55]). Pairs involving BMI-based criteria showed either “no agreement” or “slight agreement” with the other methods, regardless of the muscle strength indicator used (κ < 0.20 or negative values).

**Table 3:** Agreement between different diagnostic criteria for sarcopenia and sarcopenic obesity in older adults. Alagoas, 2022–2024.

Condition	Diagnostic combinations compared	Kappa (95% IC)	PPA (%) (95% IC)	NPA (%) (95% IC)	Kappa level of agreement
Sarcopenic					
S1 vs.	S2	0.48 (0.39–0.55)	53.9 (46.1–61.3)	92.6 (91.1–93.9)	Moderate
SO					
SO1 vs.	SO2	−0.00 (−0.01 to −0.00)	0.0 (0.0–0.0)	99.7 (99.2–99.8)	Slight
	SO4	−0.00 (−0.01 to 0.00)	0.0 (0.0–0.0)	97.5 (96.6–98.1)	No agreement
	SO5	0.13 (0.03–0.37)	13.3 (0.0–37.5)	98.5 (97.8–99.0)	Slight
	SO6	−0.00 (−0.01 to 0.00)	0.0 (0.0–0.0)	97.2 (96.2–97.9)	No agreement
SO2 vs.	SO4	0.17 (0.05–0.36)	17.8 (5.0–36.7)	97.8 (97.0–98.4)	Slight
	SO5	−0.01 (−0.02 to −0.00)	0.0 (0.0–0.0)	98.1 (97.3–98.7)	No agreement
	SO6	0.16 (0.04–0.33)	16.3 (4.7–34.5)	97.6 (96.7–98.2)	Slight
SO4 vs.	SO5	0.26 (0.13–0.42)	29.0 (16.0–44.2)	97.1 (96.1–97.8)	Fair
	SO6	0.58 (0.45–0.70)	60.5 (47.2–71.9)	97.9 (97.1–98.6)	Moderate
SO5 vs.	SO6	0.62 (0.47–0.74)	63.0 (48.5–75.0)	98.4 (97.7–98.9)	Substantial

BMI, body mass index; WC, waist circumference; %BF (BIA), body fat percentage by bioelectrical impedance analysis; HGS, hand grip strength; STS, sit-to-stand test; FFMI, fat-free mass index. Diagnostic combinations: S1 = ↓HGS + ↓FFMI; S2 = ↓STS + ↓FFMI; SO1 = ↑BMI + ↓HGS + ↓FFMI; SO2 = ↑BMI + ↓STS + ↓FFMI; SO3 = ↑WC + ↓HGS + ↓FFMI; SO4 = ↑WC + ↓STS + ↓FFMI; SO5 = ↑BF% + ↓HGS + ↓FFMI; SO6 = ↑BF% + ↓STS + ↓FFMI. PPA, positive percent agreement; NPA, and negative percent agreement.

The SO3 combination was not included in the agreement analyses because it did not identify any cases of sarcopenic obesity (*n* = 0; 0.0%). Agreement was assessed using Cohen’s Kappa coefficient and classified according to Landis and Koch (1977).

The highest agreement was observed between the two criteria that shared body fat percentage as the adiposity indicator, regardless of the functional test used: SO5 and SO6 (κ = 0.62; 95% CI = [0.47–0.74]; substantial agreement). Moderate agreement was observed for the SO4 & SO6 pair (κ = 0.58; 95% CI = [0.45–0.70]). The SO4 and SO5 pair showed fair agreement (κ = 0.26; 95% CI = [0.13–0.42]).

Across all comparisons, negative percent agreement was consistently high, whereas positive percent agreement varied substantially across diagnostic combinations, particularly among BMI-based definitions, indicating that the low kappa values observed for several comparisons were largely driven by disagreement in the identification of positive cases rather than by disagreement in negative classifications.

## 4 Discussion

To our knowledge, this study is among the first to evaluate a representative sample of community-dwelling older adults from Alagoas, Brazil, and to identify significant variability in the prevalence estimates of sarcopenia and sarcopenic obesity according to different combinations of diagnostic criteria permitted by the European EWGSOP2 consensus (1) and the ESPEN/EASO consensus statement (2). The absence of standardized diagnostic criteria with greater biological precision for these conditions represents one of the major challenges in contemporary gerontology based on precision diagnostic assessment, because the different available consensuses produce highly heterogeneous prevalence estimates, even when a single consensus is applied, given the multiple possible combinations of body composition and muscle strength markers used for diagnosis. This variability compromises the reliability of the diagnostic criteria, hinders reproducibility, and limits comparisons across studies, with a direct impact on epidemiology and on the development of public policies aligned with real-world demands.

The prevalence of sarcopenia observed in this study was approximately twice as high when the sit-to-stand test (STS) was used as the muscle strength criterion compared with handgrip strength (HGS). Similar findings have been reported by other researchers, in which the prevalence diagnosed using the STS tended to be twofold or even higher than that identified using HGS (29, 30). This substantial difference between the muscle strength tests may be related to the greater ability of the STS to identify impaired muscle strength in the lower limbs, where changes related to muscle atrophy and reductions in strength and power may occur earlier in older adults (31). In addition, the STS assesses not only muscle strength but also components of overall physical performance, such as balance, endurance, and mobility (12, 32). However, these findings are not consistent across studies. A cohort study involving Asian individuals reported results that differed from those of the present study, with a prevalence of 21% for HGS and 6.1% for STS (33). The agreement between combinations S1 and S2, classified as moderate, was similar to that reported in several studies, in which agreement ranged from low to moderate (13, 30, 34), indicating limited diagnostic overlap.

Regarding sarcopenic obesity, combinations SO1 and SO2 showed the lowest prevalence estimates, suggesting a limited ability of BMI (used as the obesity criterion) to identify excess adiposity in this population, regardless of the muscle strength criterion applied. Similar findings have been reported in studies conducted among older adults in China, in which the BMI-based criterion identified a prevalence ranging from only 0.1 to 0.63% (35, 36). It is noteworthy that the aging process is characterized by changes in body composition, including a gradual decline in body water content and muscle mass, alongside an increase in adiposity. Consequently, the body weight of some older adults may remain relatively stable despite substantial changes, rendering this indicator unable to accurately reflect these changes in body composition (37).

The SO3 combination showed no agreement with the other combinations evaluated, whereas the SO4 combination showed prevalence estimates similar to those observed for SO5 and SO6. However, agreement was only fair when SO4 was compared with the combination that used handgrip strength (HGS) as the criterion for low muscle strength (SO5) and “moderate” when the criterion was the sit-to-stand test (STS) (SO6), which may suggest that this test had a greater influence on the agreement observed between these diagnostic methods (SO4 and SO6). These findings indicate that STS was the indicator was capable of identifying a greater number of cases of low muscle strength in community-dwelling older adults and suggest that variability in the methods used may compromise the comparability of diagnostic criteria for sarcopenic obesity. A study conducted among community-dwelling older adults in Spain found higher prevalence estimates of sarcopenic obesity when waist circumference (WC) was used as the obesity criterion compared with combinations based on BMI, reinforcing the limited accuracy of BMI as a criterion for diagnosing sarcopenic obesity. These findings also suggest that central obesity combined with lower-limb strength assessment may be an important indicator of sarcopenic obesity (12), although this approach should be used with caution, particularly when only BMI and WC are available as adiposity indicators.

Additionally, the highest prevalence estimate was observed for the SO6 combination, which uses body fat percentage as the obesity criterion for the diagnosis of sarcopenic obesity, corroborating findings from other studies (36, 38, 39) that also identified higher prevalence estimates when this same criterion was adopted to identify obesity in the population. This pattern suggests that this indicator may be a more sensitive measure for detecting excess adiposity in individuals with reduced muscle strength and muscle mass. Indeed, BIA assessment, in the absence of Dual-Energy X-ray Absorptiometry (DXA), allows direct quantification of body fat, which may explain its greater ability to identify cases compared with criteria based on indirect measures such as waist circumference (40).

All combinations of adiposity criteria that used the sit-to-stand test (STS) as the muscle strength parameter showed higher prevalence estimates, reinforcing the hypothesis that this test may have a greater ability to identify reduced muscle strength for the diagnosis of sarcopenia in older adults than handgrip strength (HGS), especially among community-dwelling older adults (30).

Interdiagnostic agreement among the combinations of criteria for sarcopenic obesity ranged from “no agreement” to “substantial agreement,” indicating partial and limited diagnostic overlap and a distinct classification of a relevant proportion of individuals depending on the muscle strength criterion adopted. This finding highlights the low diagnostic equivalence among the various combinations and methods, a condition that underscoring the need for better delineation and more precise criteria for the diagnosis of these clinical conditions. Despite the limitations in interpreting the results of this study due to the absence of a gold standard for assessing body fat (such as DXA or computed tomography) and muscle function/strength (such as lower-limb electromyography), the findings expose the limitations of current consensuses and guidelines for sarcopenia and sarcopenic obesity, reinforce the hypothesis that BMI should not be used as an adiposity indicator for determining sarcopenic obesity, and suggest that HGS may represent a limited criterion whose use should be interpreted with caution (30, 36, 41).

The SO5 and SO6 combination were the only one to achieve “substantial agreement,” demonstrating that when body fat percentage is adopted as the adiposity criterion, the choice between HGS and STS as the muscle strength parameter has a limited impact on diagnosis, although a considerable proportion of individuals still were still classified differently these combinations. Overall, the results indicate that agreement tends to be higher among methods that share the same adiposity indicator (body fat percentage), use STS as the indicator of low muscle strength, and substantially lower when BMI is involved, regardless of the muscle strength parameter evaluated, a pattern consistent with the scientific literature (41–43).

It is important to emphasize that the present study was not intended to establish alternative diagnostic criteria for sarcopenic obesity or to modify the diagnostic framework proposed by the ESPEN/EASO consensus. Rather, the different adiposity indicators were examined as alternative operational definitions within an exploratory epidemiological approach to assess the impact of the selected adiposity criterion on prevalence estimates. Consequently, the observed differences should not be interpreted as evidence supporting equivalent diagnostic definitions, but instead as an illustration of the methodological variability introduced by the choice of adiposity indicator. These findings reinforce the need for standardized diagnostic criteria to improve the comparability of epidemiological studies.

Despite the relevance of this study, particularly in encouraging the scientific community and major societies in the fields of nutrition, geriatrics, and gerontology to advance toward more accurate diagnostic criteria for sarcopenia and SO, several limitations should be acknowledged, and the findings should be interpreted with caution. The cross-sectional design of the study, combined with the absence of a gold standard for the diagnosis of these conditions in this research, does not allow determination of which evaluated diagnostic combination is superior. However, identifying the best combination was not the objective of this study. Nevertheless, assuming that more sensitive methods tend to identify a greater number of individuals at risk for a given health condition and are therefore the most recommended when the goal is prevention or treatment through reducing the number of untreated false negatives, the combinations using the sit-to-stand test (STS) as an indicator of low muscle strength and body fat percentage as the adiposity criterion identified the greatest number of cases of sarcopenia and sarcopenic obesity and may therefore currently represent the most suitable alternatives. However, these findings present sufficient inferential strength to demonstrate that the wide range of available methods and criteria contributes more to the fragmentation of knowledge and to the broad variability in prevalence estimates reported across studies (even when the studied populations present similar characteristics) than to advances in the diagnosis of sarcopenia and SO.

Nevertheless, measuring and analyzing muscle assessment parameters as continuous variables would minimize information loss, including within-category variability, and improve statistical power in studies designed to assess sensitivity (44). It should be noted that the indicators used in the present study were operationalized dichotomously owing to the nature of this research. The consensus definitions evaluated (1, 2) define sarcopenia and sarcopenic obesity as binary categories, and the measures applied, including kappa, PPA and NPA, presuppose categorical classifications. Notably, the field has been moving in a different direction, with recent proposals conceptualizing sarcopenia as an advanced stage along a muscle health continuum rather than as a binary diagnosis (45), alongside recognition, by the GLIS initiative, of the need for a globally accepted conceptual definition of sarcopenia (46). Accordingly, the variability documented here should be interpreted not only as heterogeneity across criteria but also as an indication that approaches based on continuous scores or on staging, which should be explored in future analyses, may provide greater diagnostic resolution than the sequence of dichotomous classifications currently recommended.

Additionally, because this was a population-based epidemiological study with data collected through household visits, it was not feasible to assess skeletal muscle mass using DXA or computed tomography. As an alternative, the fat-free mass index estimated by BIA was used because it has been shown to correlate strongly with DXA (*r* > 0.93 across all analyzed groups) (22). To minimize potential biases associated with BIA, participants received prior instructions regarding test preparation according to the manufacturer’s recommendations, and individuals with edema were excluded from the analysis.

Nevertheless, the findings of this study should be interpreted with caution, particularly regarding the estimated prevalence of the conditions investigated. Although the adopted cut-off values represent a practical alternative for large population-based studies, especially in the absence of validated criteria for the Brazilian population, variations in body composition related to ethnicity, ageing, adiposity, and hydration status may affect the accuracy of this classification, particularly among individuals with obesity. Therefore, further studies are warranted to establish and validate population-specific cut-off values for Brazilian older adults or populations with similar demographic and anthropometric characteristics, thereby improving the diagnostic accuracy of sarcopenia in epidemiological research.

Finally, the coexistence of multiple consensuses with heterogeneous criteria has been one of the main factors contributing to imprecision in global prevalence estimates of sarcopenia and SO, making the standardization of diagnostic criteria imperative. To this end, studies using gold-standard methods for the assessment of muscle strength and adiposity are needed in order to identify which lower-cost tools present equivalent sensitivity and specificity and may therefore safely replace imaging examinations in primary healthcare settings with limited resources. Additionally, in these contexts, the adoption of a single criterion for each diagnostic component—muscle strength, muscle mass, and adiposity—is recommended, with body fat percentage estimated by BIA representing a feasible alternative in community settings when DXA or computed tomography are unavailable, as well as the five-repetition sit-to-stand test when electromyography is not feasible.

## 5 Conclusion

The prevalence estimates of sarcopenia and SO were markedly heterogeneous according to the combination of muscle strength and adiposity indicators adopted. The level of interdiagnostic agreement ranged from “no agreement” to “substantial.” From a public health perspective, the findings of this study reinforce the need to establish standardized and consistent criteria for consistent population screening of these syndromes, enabling early interventions and the consequent reduction of healthcare system costs.

## Data Availability

The raw data supporting the conclusions of this article will be made available by the authors, without undue reservation.
