# Supplementary material for: Diagnostic variability in sarcopenia and sarcopenic obesity among older adults: a clinical and public health challenge
Source: Front Nutr. 2026 Aug 27;13:1895826. doi: 10.3389/fnut.2026.1895826 (PMC13558103; doi:10.3389/fnut.2026.1895826)
Supplement: Supplementary file 1 [file Table_1.DOCX]

Supplementary Material

**Supplementary Table S1.** Body composition, muscle strength and physical performance according to sex group: descriptive statistics and between-group comparisons among Community-Dwelling Older Adults. Alagoas, Brazil, 2022–2024.

| **Variable** | **Women** | | | **Men** | | | **B** | **95%CI β** | ***p*** |
| --- | --- | --- | --- | --- | --- | --- | --- | --- | --- |
|  | **n** | **Mean ± SD** | **95%CI** | **n** | **Mean ± SD** | **95%CI** |  |  |  |
| ***Body composition*** |  |  |  |  |  |  |  |  |  |
| BMI (kg/m²) | 574 | 28.18 ± 5.58 | 27.72 to 28.64 | 296 | 26.53 ± 4.87 | 25.97 to 27.08 | -1.66 | -2.38 to -0.94 | <0.001 |
| WC (cm) | 574 | 95.74 ± 13.44 | 94.63 to 96.85 | 296 | 95.80 ± 13.03 | 94.30 to 97.30 | 6.06 | -1.80 to 1.92 | 0.947 |
| % BF (%) | 574 | 37.00 ± 7.91 | 36.35 to 37.65 | 296 | 25.37 ± 8.17 | 24.44 to 26.30 | -11.63 | -12.76 to -10.49 | <0.001 |
| FFMI (kg/m²) | 574 | 16.55 ± 1.95 | 16.39 to 16.71 | 296 | 18.68 ± 2.41 | 18.40 to 18.95 | 2.13 | 1.81 to 2.45 | <0.001 |
| ***Muscle strength and physical performance*** |  |  |  |  |  |  |  |  |  |
| HGS (kg) | 574 | 18.90 ± 6.33 | 18.38 to 19.42 | 296 | 30.20 + 8.14 | 29.27 to 31.13 | 11.30 | 10.24 to 12.36 | <0.001 |
| TUG (s) | 420 | 11.17 ± 4.64 | 10.73 to 11.62 | 230 | 10.29 + 4.66 | 9.68 to 10.89 | -0.89 | -1.63 to -0.14 | 0.020 |
| GST (m/s) | 406 | 1.23 ± 0.72 | 1.16 to 1.30 | 218 | 1.06 + 0.40 | 1.01 to 1.11 | -0.17 | -0.26 to -0.08 | <0.001 |
| STS (s) | 487 | 19.98 ± 7.98 | 19.27 to 20.70 | 258 | 17.42 + 5.03 | 16.81 to 18.04 | -2.56 | -3.50 to -1.62 | <0.001 |

**Caption** - BMI: body mass index; WC: waist circumference; % BF: body fat percentage by bioelectrical impedance analysis; FFMI: fat-free mass index; HGS: hand grip strength; TUG: Timed Up and Go test; GST: Gait Speed Test; STS: sit-to-stand test with time.

**Note** - Values are presented as mean ± standard deviation and 95% confidence interval. Between-group comparisons were performed using simple linear regression with heteroskedasticity-robust standard errors. The β coefficient represents the mean difference between women and men older adults.

**Supplementary Table S2.** Body composition, muscle strength, and physical performance according to age group (long-lived and non-long-lived older adults): descriptive statistics and between-group comparisons among community-dwelling older adults. Alagoas, Brazil, 2022–2024.

| **Variable** | **Non-long-lived** | | | **Long-lived** | | | **B** | **95%CI β** | ***p*** |
| --- | --- | --- | --- | --- | --- | --- | --- | --- | --- |
|  | **n** | **Mean ± SD** | **95%CI** | **n** | **Mean ± SD** | **95%CI** |  |  |  |
| ***Body composition*** |  |  |  |  |  |  |  |  |  |
| BMI (kg/m²) | 728 | 27.77 ± 5.46 | 27.38 to 28.17 | 142 | 26.75 ± 5.01 | 25.94 to 27.61 | -1.00 | -1.92 to -0.08 | 0.033 |
| WC (cm) | 728 | 95.67 ± 13.55 | 94.68 to 96.66 | 142 | 96.22 ± 11.91 | 94.23 to 98.21 | 0.55 | -1.67 to 2.76 | 0.628 |
| % BF (%) | 728 | 33.14 ± 9.67 | 32.44 to 33.85 | 142 | 32.46 ± 9.96 | 30.79 to 34.12 | -0.69 | -2.48 to 1.11 | 0.456 |
| FFMI (kg/m²) | 728 | 17.34 ± 2.35 | 1717 to 17.52 | 142 | 16.94 ± 2.29 | 16.56 to 17.32 | -0.40 | -0.81 to 0.02 | 0.059 |
| ***Muscle strength and physical performance*** |  |  |  |  |  |  |  |  |  |
| HGS (kg) | 728 | 23.53 ± 8.83 | 22.89 to 24.17 | 142 | 18.89 ± 7.73 | 17.61 to 20.18 | -4.64 | -6.07 to -3.20 | <0.001 |
| TUG (s) | 558 | 10.36 ± 4.38 | 10.00 to 10.73 | 92 | 13.87 ± 5.20 | 12.79 to 14.95 | 3.51 | 2.38 to 4.63 | <0.001 |
| GST (m/s) | 535 | 1.13 ± 0.64 | 1.07 to 1.18 | 89 | 1.45 ± 0.54 | 1.33 to 1.56 | 0.32 | 0.19 to 0.45 | <0.001 |
| STS (s) | 644 | 18.63 ± 6.90 | 18.10 to 19.17 | 101 | 22.11 ± 8.30 | 20.46 to 23.77 | 3.48 | 1.75 to 5.21 | <0.001 |

**Caption** - BMI: body mass index; WC: waist circumference; % BF: body fat percentage by bioelectrical impedance analysis; FFMI: fat-free mass index; HGS: hand grip strength; TUG: Timed Up and Go test; GST: Gait Speed Test; STS: sit-to-stand test with time.

**Note** - Values are presented as mean ± standard deviation and 95% confidence interval. Between-group comparisons were performed using simple linear regression with heteroskedasticity-robust standard errors. The β coefficient represents the mean difference between long-lived and non-long-lived older adults.
